# [^18^F]FDG-PET/CT texture analysis in thyroid incidentalomas: preliminary results

**DOI:** 10.1186/s41824-017-0009-8

**Published:** 2017-10-12

**Authors:** M. Sollini, L. Cozzi, G. Pepe, L. Antunovic, A. Lania, L. Di Tommaso, P. Magnoni, P. A. Erba, M. Kirienko

**Affiliations:** 1 Department of Biomedical Sciences, Humanitas University, via Rita Levi Montalcini, 20090 Pieve Emanuele (Milan), Italy; 2 Radiotherapy and Radiosurgery, Humanitas Clinical and Research Center, via Manzoni 56, 20089 Rozzano (Milan), Italy; 3 Nuclear Medicine, Humanitas Clinical and Research Center, via Manzoni 56, 20089 Rozzano (Milan), Italy; 4 Endocrinology, Humanitas Clinical and Research Center, via Manzoni 56, 20089 Rozzano (Milan), Italy; 5 Pathology, Humanitas Clinical and Research Center, via Manzoni 56, 20089 Rozzano (Milan), Italy; 6 Ultrasound Service, Humanitas Clinical and Research Center, via Manzoni 56, 20089 Rozzano (Milan), Italy; 7 Regional Center of Nuclear Medicine, University of Pisa, via Roma 55, 56025 Pisa, Italy

**Keywords:** Thyroid, Incidentaloma, [^18^F]FDG-PET/CT, Texture analysis, Radiomics

## Abstract

**Background:**

significance of incidental thyroid 2-deoxy-2-[^18^F]fluoro-D-glucose ([^18^F]FDG) uptake on positron emission tomography/computed tomography (PET/CT) scans remains controversial. We aimed to evaluate the ability of [^18^F]FDG-PET/CT texture analysis to predict final diagnosis in thyroid incidentaloma.

**Methods:**

We retrospectively evaluated medical records of all patients who performed a [^18^F]FDG-PET/CT from January 2012 to October 2016. Those patients who presented a thyroid incidentaloma described in the medical records and performed a fine needle aspiration in our institution were considered for the analysis. Cytological and/or histological results were used as reference standard to define the final diagnosis. In case of negative cytology, the nodule was considered benign. In case of non-diagnostic or inconclusive results ultrasound, follow-up and further cytology/histology were used as final diagnosis. For suspected or positive cytological result, histology was used as reference standard. PET images were segmented using a General Electric AW workstation running PET VCAR software (GE Healthcare, Waukesha, WI, USA) settled with a threshold of 40% SUV_max_. LifeX software (http://www.lifexsoft.org) was used to perform texture analysis. Statistical analysis was performed with R package (https://www.r-project.org).

**Results:**

We identified 55 patients with incidental thyroid [^18^F]FDG uptake. Five patients were excluded from the analysis because a final diagnosis was not available. Thirty-two out of 50 patients had benign nodules while in 18/50 cases a malignancy (primary thyroid cancer = 15, metastases = 3) was diagnosed. Conventional PET parameters and histogram-based features were calculated for all 50 patients, while other matrices-based features were available for 28/50 patients. SUV_max_ and skewness resulted significantly different in benign and malignant nodules (*p* = 0.01 and = 0.02, respectively). Using ROC analysis, seven features were identified as potential predictors. Among all the textural features tested, skewness showed the best area under the curve (= 0.66). SUV-based parameters resulted in the highest specificity while MTV, TLG, skewness and kurtosis, as well as correlation_GLCM_ resulted better in sensitivity.

**Conclusions:**

[^18^F]FDG-PET/CT texture analysis seems to be a promising approach to stratify the patients with thyroid incidentaloma identified on PET scans, with respect to the risk of the diagnosis of a malignant thyroid nodule and thus, could refine the selection of the patients to be referred for cytology.

**Electronic supplementary material:**

The online version of this article (10.1186/s41824-017-0009-8) contains supplementary material, which is available to authorized users.

## Background

Incidentalomas are findings on an imaging test performed for other reasons, for which there are no matching symptoms in the patient (Davies et al. 2016). Published reports estimated the prevalence of thyroid incidentalomas on [^18^F]FDG-PET images between 1.2% and 4.3% (Rigo et al. 1996; Mitchell and Parangi 2005; Liu et al. 2010). These data are in line with a recent meta-analysis which reported a prevalence of thyroid incidentalomas of 1.8% (3659 cases out of 197,296 [^18^F]FDG-PET/CT examinations), of which a third (36.6%) were evaluated with cytopathology or histopathology (Nayan et al. 2014). Although the overall incidence of thyroid incidentalomas detected on PET imaging is low, the chance of malignancy among incidentalomas detected by [^18^F]FDG is higher than incidental nodules discovered with other imaging modalities, with a chance for malignancy ranging from 14 to 59% (Gavriel et al. 2015; Lee et al. 2014; Demir et al. 2016; Chun et al. 2015; Are et al. 2007; Pagano et al. 2011; Cohen et al. 2001; Nilsson et al. 2011). Nonetheless, we have a limited understanding of the incidentaloma natural history (Davies et al. 2016). Therefore, the differentiation between malignant and benign thyroid lesions is crucial to avoid unnecessary procedures, to improve the quality of life of patients, and to reduce healthcare costs.

Many researchers postulated that malignant lesions tend to show higher [^18^F]FDG uptake (i.e., SUV_max_) than that of benign lesions (Soelberg et al. 2012). However, it is not easy to differentiate malignancy from benignity using maximum standardized uptake value (SUV_max_) only (Demir et al. 2016; Chun et al. 2015). Some authors proposed the evaluation of other parameters in addition to SUV_max_ of the thyroid incidentaloma, such as the target/background, the target/blood-pool, and the target/liver ratios (Barrio et al. 2016). Also the pattern of [^18^F]FDG uptake (focal versus diffuse) together with the elastography score (≥ 4) and some ultrasound findings (echogenicity, spot microcalcifications, color-flow Doppler pattern) have been tested (Demir et al. 2016). Dual time point PET imaging has been proposed as alternative method to overcome the low specificity of SUV_max_ in the differentiation of benign from malignant lesions, including thyroid incidentalomas (Lee et al. 2014). However, all these approaches were not validated and, they are not generally accepted. Therefore, fine-needle aspiration (FNA) remains the procedure of choice to define the diagnosis. If the nodule is benign on cytology, further immediate diagnostic studies or treatment are not required; while if cytology results diagnostic for malignancy, surgery is generally recommended. For a nodule with a non-diagnostic cytology result, FNA should be repeated (Haugen et al. 2016).

In the past years, interest has grown in texture analysis of medical images that provide numerous quantitative and semi-quantitative parameters capturing the inhomogeneity of the tissues. This analysis seems able to provide a better characterization of cancer lesions, and some prognostic information about the aggressiveness of disease using conventional medical imaging (Buvat et al. 2015). No data on [^18^F]FDG-PET/CT images texture analysis in thyroid have been reported in the literature, with the exception of Kim et al. (Kim and Chang 2015). They evaluated some parameters, including a feature named “heterogeneity factor”, derived from the histogram of intensities of uptake within the lesion, in patients with a thyroid nodule (Kim and Chang 2015).

The aim of this study was to investigate the ability of texture analysis on [^18^F]FDG-PET/CT to predict final diagnosis in thyroid incidentalomas.

## Methods

### Patients

We retrospectively evaluated the medical records of all the patients who performed a [^18^F]FDG-PET/CT from January 2012 to October 2016. Those patients who presented thyroid [^18^F]FDG uptake (described in the medical record) and performed a cytological examination in our institution were considered for the analysis. According to good clinical practice in use in our institution, cytological examination was performed in case of doubtful or suspected ultrasound findings (i.e., solid nodule, microcalcifications; hypoechogenicity; increased nodular vascularity; irregular or infiltrative margins; taller than wide on transverse view).

Demographics, data relating to [^18^F]FDG-PET/CT scan and its findings, cytological results, biochemical data, surgery details and histopathology (when available) were reviewed. Among all subjects who underwent a [^18^F]FDG-PET/CT examination (17,104 scans) and had a thyroid uptake on the images (453 subjects), we identified 55 patients (male:female = 20:35; age 62 ± 15 years) that performed a cytological examination in our institution. Indication for [^18^F]FDG-PET/CT was oncological in 50 cases and non-oncological in the remaining 5 cases (2 vasculitis, 1 sarcoidosis, 1 fever of unknown origin, 1 suspected orthopedic infection). In the oncologic group, [^18^F]FDG-PET/CT was performed for diagnosis or staging in 26 cases, re-staging in 14 cases, and follow-up in 10 cases.

Ultrasound-guided FNA was performed according to the standard procedure in use in our institution. Cytological results were reported according to the SIAPEC-IAP 2007 classification, and grouped in 5 diagnostic categories defined as TIR1 or non-diagnostic, TIR2 or negative for malignant cells, TIR3 or inconclusive/indeterminate, TIR4 or suspicious for malignancy, TIR5 or diagnostic of malignancy (i.e., papillary, medullary and anaplastic carcinomas, lymphomas and metastasis) (Fadda et al. 2010). All cytological results reported according the update version of the SIAPEC-IAP 2014 classification (Nardi et al. 2014), were reclassified for the present analysis using the SIAPEC-IAC 2007 classification. Cytology and/or histology was used as reference standard to define the final diagnosis. In case of negative cytology (TIR2), no further studies were immediately performed, and the nodule was considered benign. In case of non-diagnostic (TIR1) or doubtful (TIR3) results, ultrasound follow-up and further cytological samples or histology were used as final diagnosis. In the case of suspected (TIR4) or positive (TIR5) cytological result, histology was used as reference standard. All cases resulted as TIR1 (*n* = 1) or TIR3 (*n* = 4) in which a further cytology/histology was not available (3 patients lost at follow-up and 2 patients had progressive disease for breast cancer and multiple myeloma, respectively), were excluded from the analysis, therefore 50 patients (male:female = 18:32; age 63 ± 15 years) were overall included. This retrospective observational study was reviewed and approved by the Local Ethics Committee.

### [^18^F]FDG PET/CT acquisition protocol

[^18^F]FDG-PET/CT image acquisition was performed according to the European Association of Nuclear Medicine (EANM) guidelines (Boellaard et al. 2014). Images were acquired 60 ± 5 min after [^18^F]FDG administration, in the fasting state using an integrated PET/CT scanner, either a Siemens Biograph LS 6 scanner (Siemens, Munich, Germany) equipped with LSO crystals and a six-slice CT scanner, or a GE Discovery PET/CT 690 equipped with LYSO crystals and a 64-slice CT scanner (General Electric Healthcare, Waukesha, WI, USA). Both scanners are EARL certified (http://www.eanm.org) and images were processed in order to minimize differences between semi-quantitative evaluation.

### Image analysis

Whole-body [^18^F]FDG-PET/CT images were interpreted visually by two experienced nuclear medicine physicians in consensus. Thyroid incidentaloma was defined as focal thyroid uptake (i.e., increased [^18^F]FDG uptake compared to surrounding or contralateral thyroid parenchyma) identified on [^18^F]FDG-PET/CT study incidentally and was described as unilateral (i.e., occurred in less than one lobe) and bilateral (i.e., involving both thyroid lobes) according to the thyroid uptake pattern.

PET images were segmented using a General Electric AW workstation running PET VCAR software (GE Healthcare, Waukesha, WI, USA) settled with a threshold of 40% SUV_max_. The region of interest (ROI), set in the target tissue (i.e., incidentaloma), was saved in 3D. Segmented images were saved in DICOM-RT format together with the corresponding [^18^F]FDG-PET/CT images. The entire dataset was then analyzed to extract a number of textural features by means of the LifeX package (http://www.lifexsoft.org) (Orlhac et al. 2016). LifeX was set up using the following input parameters for calculation of features: 64 Gy levels to resample the ROI content which was performed in absolute terms between a minimum of 0 and a maximum of 20 (Orlhac et al. 2015). A total of 43 features were extracted from the analysis of the volumes inspected. These indices included conventional parameters, shape and size features, histogram-based features, second and high order-based features. The correction for the partial volume effect was not applied. In the analysis were included all incidentalomas, irrespectively of their volume but LifeX calculates the shape and size indices as well as second order (gray-level co-occurrence matrix) and high order-based (neighborhood gray-level different matrix, gray-level run-length matrix, and gray-level zone-length matrix) features only for ROI of at least 64 voxels due to technical reasons. The features calculated are summarized in Table 1. For the features resulted significant, the formula is provided.

**Table 1:** Texture features calculated from the [^18^F]FDG-PET/CT images in Lifex to characterize thyroid incidentalomas

Features	Formulas for features resulted significant
Gray-level co-occurrence matrix (GLCM)	
Homogeneity 1 Energy_GLCM_ Contrast_GLCM_ Correlation_GLCM_ Entropy_GLCM_ Dissimilarity	Correlation measures the linear dependency of the gray levels in the GLCM matrix and is defined as the average over the 13 directions of: $\left(\sum_{i,j}\frac{\left(i-\mu \right)x\left(j-\mu \right) xC\left(i,j\right)}{\sigma_i{\sigma}_j}\right)$ where μ_i,j_ correspond to the average on row i or column j and σ_i,j_ correspond to the variance on row i or column j.
Neighborhood gray-level different matrix (NGLDM)	
Contrast_NGTDM_ Coarseness	
Gray-level run-length matrix (GLRLM)	
Short-Run Emphasis Long-Run Emphasis Low Gray-level Run Emphasis High Gray-level Run Emphasis Short-Run Low Gray-level Emphasis Short-Run High Gray-level Emphasis Long-Run Low Gray-level Emphasis Long-Run High Gray-level Emphasis Gray-Level Non-Uniformity for run Run Length Non-Uniformity Run Percentage	
Gray-level zone-length matrix (GLZLM)	
Short-Zone Emphasis Long-Zone Emphasis Low Gray-level Zone Emphasis High Gray-level Zone Emphasis Short-Zone Low Gray-level Emphasis Short-Zone High Gray-level Emphasis Long-Zone Low Gray-level Emphasis Long-Zone High Gray-level Emphasis Gray-Level Non-Uniformity for zone Zone Length Non-Uniformity Zone Percentage	
Shape and Size	
Sphericity Compacity	Compacity (Shape and size): $\frac{A^{3/2}}{V}$ where A and V correspond to the area and the volume of the volume of interest from the Delaunay triangulation.
Histogram	
Skewness Kurtosis Entropy_Hist_ Energy_Hist_	Skewness $\frac{a\sum_i{\left(H(i)- Mean\right)}^3}{{\left(\sqrt{a\sum_i{\left(H(i)- Mean\right)}^2}\right)}^3}$ where a is the inverse of the total number of voxels in the volume of interest and mean is the average of the intensity values in the histogram. The sum is extended to all voxels in the volume of interest. Kurtosis $\frac{a\sum_i{\left(H(i)- Mean\right)}^4}{{\left(a\sum_i{\left(H(i)- Mean\right)}^2\right)}^2}$
Conventional parameters	
SUV minimum, SUV maximum, SUVmean and SUVstandard deviation, SUVpeak within a sphere of 0.5 and 1 ml volume (mL) Total lesion glycolysis (TLG)	SUV_max_ maximum of the standardized uptake value in the volume of interest SUV_std_ standard deviation of the SUV distribution in the volume of interest MTV the metabolic tumor volume was determined as the total number of voxels with SUV > 40% TLG is defined as the product of SUV_mean_ times the volume

The gray-level co-occurrence matrix (GLCM) was calculated from 13 different directions in 3D with a 1-voxel distance relationship between consecutive voxels

The neighborhood gray-level different matrix (NGLDM) corresponds to the difference of gray level between one voxel and its 26 neighborhoods in 3 dimensions

The gray-level run-length matrix (GLRLM) gives the size of homogeneous runs for each gray level. This matrix is computed in 13 different directions in 3 dimensions

The gray-level zone-length matrix (GLZLM) provides information on the size of homogeneous zones for each gray level in 3 dimensions

Histogram represents the gray level distribution within the volume of interest

Skewness - measure of the asymmetry of the distribution, kurtosis - measuring weather the distribution is peaked or flat relative to a normal distribution, Entropy_Hist_ - randomness of the distribution, Energy_Hist_ - uniformity of the distribution

### Statistical analysis

Statistical analysis was performed using the open source R platform (https://www.r-project.org). Continuous normally distributed data were expressed as mean **±** standard deviation. Conventional PET parameters and textural features were compared to SIAPEC-IAP categories and final diagnosis, using the Anova test. A *p*-value < 0.05 was considered statistically significant.

Receiver operating characteristic (ROC) curves for each parameter were determined and the corresponding area under the curve (AUC) computed for each of those. A preliminary selection of the predictors was computed excluding those with an AUC < 0.55. The mutual correlation between features was evaluated for those metrics, preliminarily selected, in order to assess potential result redundancy.

Among the pre-selected predictors, a further analysis was performed to identify those with statistically significant (or with a tendency to significance) capability to stratify between positive (malignant) and negative (benign) cases. The dichotomization was performed by means of an iterative process aiming to identify the predictor’s threshold minimizing the p-value of the Fisher’s test. The final predictors were selected if *p* < 0.08.

For each predictor in the final list, the truth table (true and false positive and negative values) was determined at the optimal cutoff value and from this the standard predictive scores were computed.

## Results

Table 2 summarizes the descriptive characteristics of the cohort of the patients included in the analysis.

**Table 2:** Descriptive statistics of the cohort of patients in the study

Parameter	Group	Number of cases	Relative %
FNA results	TIR1	2	4.0
	TIR2	26	52.0
	TIR3	7	14.0
	TIR4	4	8.0
	TIR5	11	22.0
Final diagnosis	Negative	32	64.0
	Positive	18	36.0
Histological type^a^	DTC	13	26.0
	PDTC	1	2.0
	MTC	1	2.0
	Lymphoma	1	2.0
	Metastases	2	4.0

*FNA* fine needle aspiration, *DTC* differentiated thyroid cancer, *PDTC* poorly differentiate thyroid cancer, *MTC* medullary thyroid cancer

^a^Only for patients with a final diagnosis positive for malignancy (*n* = 18)

FNA was non-diagnostic in 2 cases, negative in 26 cases, doubtful in 7 cases, suspected in 4 cases, and positive for malignant tumor cells in 11 cases. Patients classified as TIR1, repeated the FNA which resulted negative (TIR2) in both cases. Therefore, considering the re-classification of TIR1 cases, the number of patients belonging to the TIR2 category increased overall to 28. Among the TIR3 cases, 3 were positive and 4 were negative (follicular adenoma = 2, oxyphil cell adenoma = 1, and colloid cystic goiter = 1); while all TIR4 or TIR5 were proven to be malignant. Nodules resulted malignant (*n* = 18) included 15 thyroid cancers (papillary thyroid cancer = 13, poor differentiated thyroid cancer n = 1, medullary thyroid cancer = 1), 2 metastases from bladder cancer and breast cancer respectively, and 1 lymphoma.

Biochemical data are summarized in Table 3.

**Table 3:** Summary of biochemical data

Biochemical parameter	Benign nodule	Malignant nodule	Overall
Thyroid stimulating hormone (mIU/L)	2.33 ± 3.35	1.76 ± 2.16	2.10 ± 2.90
Anti-thyroglobulin antibodies^a^	Positive = 5	Positive = 4	Positive = 9
	Negative = 16	Negative = 7	Negative = 23
Anti-thyroid peroxidase antibodies^a^	Positive = 8	Positive = 4	Positive = 12
	Negative = 13	Negative = 7	Negative = 20
Calcitonin (ng/L)^b^	1.89 ± 1.62	5.70 ± 7.50	3.39 ± 5.10

^a^Available in 32/50 patients

^b^Available in 28/50 patients

Thyroid stimulating hormone (TSH) resulted lower than the low normal limit (< 0.25 mIU/L) in 5 cases (3 benign and 2 malignant). High TSH value (> 4.2 mIU/L, i.e., higher than the upper normal limit) was observed in 4 cases (3 benign and 1 malignant). Anti-thyroid peroxidase antibodies (TPOAb), anti-thyroglobulin antibodies (TgAb), and calcitonin (hCT) were not available for all the patients (Table 2). hCT resulted abnormal in 2/28 cases (both diagnosed as papillary thyroid cancer), while it was normal in the remaining 26/28 cases (including the patient with medullary thyroid cancer).

**Fig. 1:**
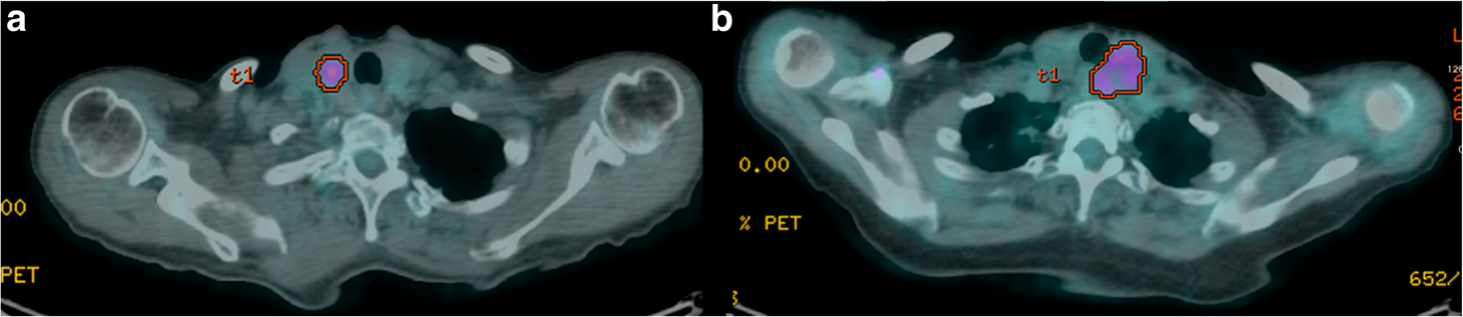
Examples of pattern of [^18^F]FDG uptake at PET/CT images of 2 thyroid incidentalomas identified by the region of interest (red circle). Moderate [^18^F]FDG uptake was observed in a benign nodule (TIR2 at cytology) of the right thyroid lobe (**a**) and in a differentiated thyroid cancer (TIR3 at cytology) of the left thyroid lobe (**b**)

**Fig. 2:**
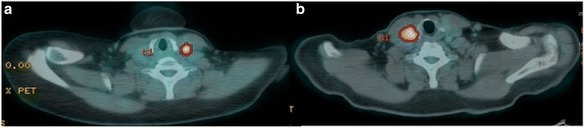
Examples of pattern of [^18^F]FDG uptake at PET/CT images of 2 thyroid incidentalomas identified by the region of interest (red circle). Intense [^18^F]FDG uptake was observed in a benign nodule (TIR3 at cytology) of the left thyroid lobe (**a**) and in a differentiated thyroid cancer (TIR5 at cytology) of the right thyroid lobe (**b**)

Thyroid [^18^F]FDG uptake was unilateral in the majority of patients (94%) occurring in the right and the left lobe in 24 and 23 cases, respectively. In the remaining 3 cases (6%) thyroid [^18^F]FDG uptake involved both lobes. Figures 1 and 2 shows examples of [^18^F]FDG-PET/CT findings in patients diagnosed as having benign and malignant thyroid nodule. Conventional PET parameters, and histogram-based features were calculated for all patients. Twenty-two patients had small ROI (i.e., < 64 voxels), therefore features extracted from shape and size, GLCM, GLZLM, NGLDM, and GLRLM were available only for 28/50 patients (TIR2 = 15, TIR3 = 4, TIR4 = 2, TIR5 = 7). According to the final diagnosis, 12 out of 28 patients for whom all features were available, resulted positive for malignancy, while in the remaining 16 cases the nodule was diagnosed as benign.

Additional file 1: Table S1 summarizes conventional PET parameters and textural features tabulated according to FNA categories and final diagnosis. Compacity (shape and size-based) was the only feature statistically significant to differentiate the TIR categories (*p* = 0.03). SUV_max_ and skewness were significantly different in benign and malignant nodules (*p* = 0.01 and =0.02, respectively).

Table 4 summarizes the 7 final features identified as potential predictors (SUVstd, SUVmax, MTV, TLG, Skewness, Kurtosis, Correlation_GLCM_) with their mean values, the AUC, the best threshold for the dichotomization of the cohort and the final *p* value.

**Table 4:** Summary of statistically significant predictors

Predictor	Mean ± St.dev	AUC [95% CI]	Best dichotomizing threshold	p
*Conventional*				
SUV_std_	1.6 ± 1.8	0.59 [0.42–0.77]	2.11	0.02
SUV_max_	9.0 ± 8.7	0.60 [0.42–0.77]	10.21	0.03
MTV [mL]	27.0 ± 94.5	0.66 [0.50–0.81]	1.52	0.03
TLG [mL]	309.5 ± 1881.7	0.66 [0.49–0.82]	9.71	0.02
*Histogram-based*				
Skewness	0.61 ± 00.52	0.66 [0.48–0.82]	0.47	0.008
Kurtosis	2.99 ± 1.69	0.55 [0.38–072]	1.90	0.07
*GLCM-based*				
Correlation	0.51 ± 0.15	0.57 [0.36–0.79]	0.35	0.05

*AUC* area under curve, *MTV* metabolic tumor volume, *TLG* total lesion glycolysis, *GLCM* gray-level co-occurrence matrix, *SUV* standardized uptake valu

**Fig. 3:**
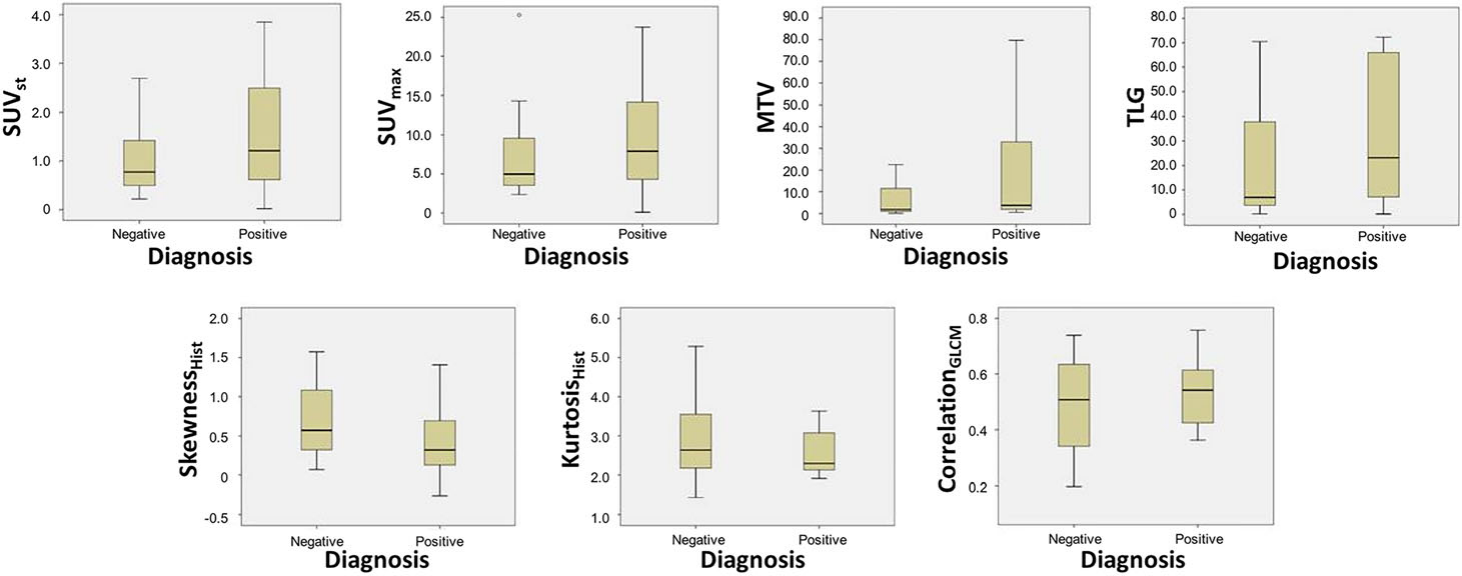
The box-plots of the 7 final indices stratified for the two subgroups (negative versus positive) in the cohort

**Fig. 4:**
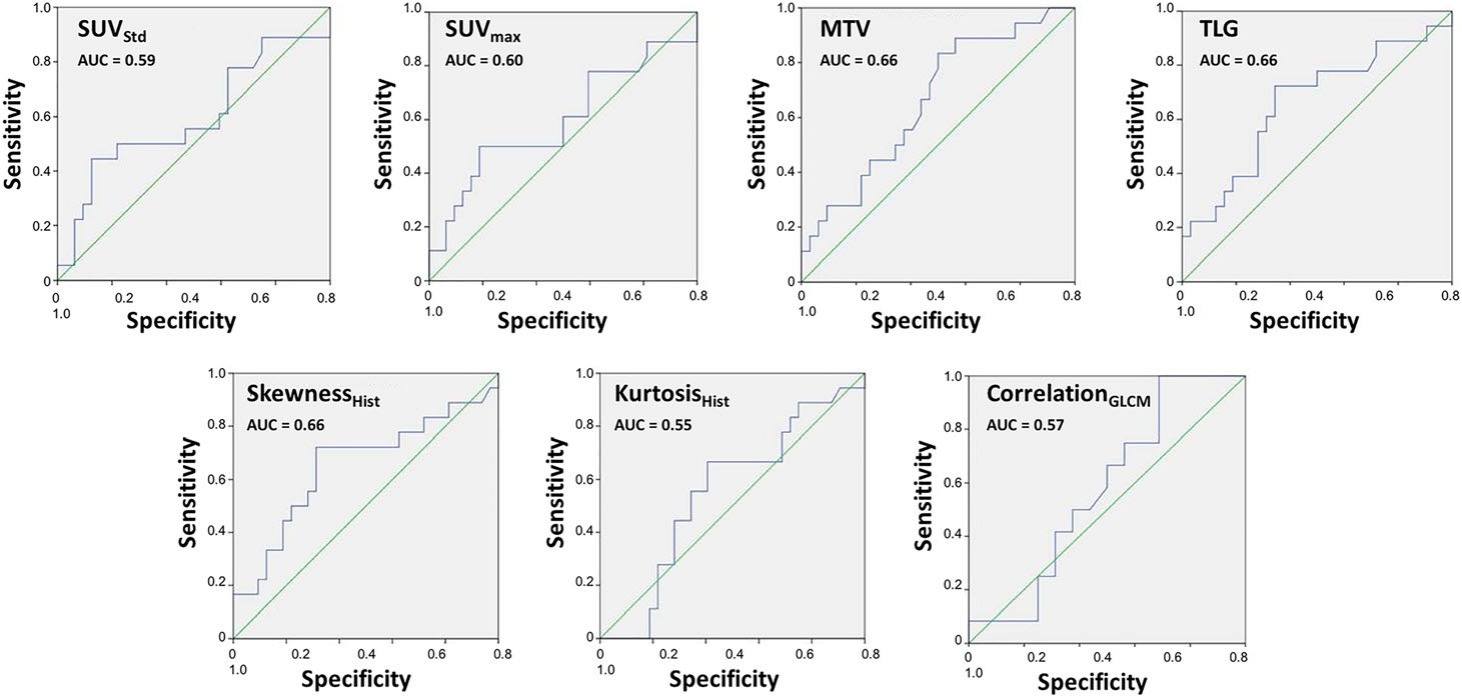
The ROC curves (together with the reference 50% AUC line) for the conventional parameters (upper panel) and the texture related features (bottom panel)

Figure 3 shows the box-plots of the 7 final features stratified for the two subgroups in the cohort. Figure 4 shows the ROC curves (together with the reference 50% AUC line) for the volume and the texture related features. Among the potential predictors a mutual correlation was found between SUV_std_ and SUV_max_ (0.967), TLG and MTV (0.970), as well as skewness and kurtosis (0.830), as shown in Table 5.

**Table 5:** Summary of the mutual correlation between features pre-selected as significant predictors

Feature	SUV_std_	SUV_max_	MTV	TLG	Skewness	Kurtosis	Correlation_GLCM_
SUV_std_	1	0.967 (^a^)	0.250	0.309	−0.123	−0.123	0.257
SUV_max_	0.967 (^a^)	1	0.438	0.491	−0.113	−0.03	0.336
MTV [mL]	0.250	0.438	1	0.970 (^a^)	−0.211	0.027	0.332
TLG [mL]	0.309	0.491	0.970 (^a^)	1	−0.240	−0.052	0.342
Skewness	−0.123	−0.113	−0.211	−0.240	1	0.830 (^a^)	0.027
Kurtosis	−0.123	−0.03	0.027	−0.052	0.830 (^a^)	1	0.110
Correlation_GLCM_	0.257	0.336	0.332	0.342	0.047	0.110	1

*MTV* metabolic tumor volume, *TLG* total lesion glycolysis, *GLCM* gray-level co-occurrence matrix, *SUV* standardized uptake value

(^a^) correlation is significant at 0.01 level

None of the features which presented a mutual correlation were excluded from further analysis.

Table 6 summarizes the predictors and their power in the identification of the negative or positive subgroups in the cohort. SUV_std_ and SUV_max_ showed the highest specificity while MTV, TLG, skewness and kurtosis, as well as correlation_GLCM_ resulted better in sensitivity.

**Table 6:** Summary of predictive power of the significant predictors

Predictor	Specificity [%]	Sensitivity [%]	Positive predictive value [%]	Negative predictive value [%]
*Conventional*				
SUV_std_^a^	88	44	67	74
SUV_max_^a^	81	50	60	74
Volume [mL]^b^	44	89	47	88
TLG [mL]^b^	66	72	54	81
*Histogram-based*				
Skewness^c^	69	72	57	81
Kurtosis^c^	19	100	41	100
*GLCM-based*				
Correlation	31	100	52	100

*SUV* standardized uptake value, *TLG* total lesion glycolysis, *GLCM* gray-level co-occurrence matrix

^a^,^b^,^c^ Pairs of features whit a mutual correlation

The average positive predictive value of the 7 features resulted 54% while the average negative predictive value 85%.

## Discussion

The current study, although in a limited number of patients, showed that PET-derived metrics could be used to characterize incidentalomas. Both SUV_max_ (conventional parameter) and skewness (histogram-based feature) differed significantly in benign and malignant thyroid nodules. In our cohort, we report prevalence of thyroid incidentaloma in line with literature data (2.6% versus 1.2–4.3%) (Rigo et al. 1996; Mitchell and Parangi 2005; Liu et al. 2010), with a low rate (i.e., < 55% (Gavriel et al. 2015)) of additional workup (ultrasound and cytology available in 19% and 12% of subjects, respectively), but a relatively high rate of malignancy (36%).

Our results confirmed data reporting that malignant nodules had higher [^18^F]FDG uptake (SUV_max_) than those resulted benign (Gavriel et al. 2015; Demir et al. 2016; Chun et al. 2015; Kim and Chang 2015). However, although SUV_max_ is the conventional PET parameter most used in clinical practice, there are several sources of bias and variability including patients’ status (e.g., blood glucose level, impaired renal function), that affect its measurement (Kinahan and Fletcher 2010; Huang 2000).

Radiomics is a post-processing approach correlating image texture parameters to clinical, genetic and prognostic variables, and it is applicable to all types of images. Time for texture processing varies among software used for both images segmentation and features extraction. In this series of patients we spent few minutes from thyroid nodule segmentation (about 3 min/patient including also time to retrieve and download images) to features extraction (about 2 min/patient depending on ROI volume). Once texture analysis tool will be implemented in the workstation used for clinical purpose, texture features extraction will be faster as it is currently for conventional parameters. Texture analysis of [^18^F]FDG-PET/CT images provides quantitative parameters describing tissue metabolic heterogeneity and seems to perform better than the conventional PET metrics in several malignances, but data on [^18^F]FDG-PET/CT radiomics in thyroid nodules are limited. Kim et al. (Kim and Chang 2015) investigated the intratumoral heterogeneity of [^18^F]FDG uptake, defined as the heterogeneity factor (HF) obtained from the derivative (dV/dT) of a volume-threshold function – a first order statistical-based approach. The authors found that HF represented a promising method for prediction of malignant thyroid nodule in 200 patients with [^18^F]FDG-PET/CT incidentaloma (Kim and Chang 2015). The global tumor heterogeneity may be described by using first order histogram-based features; the second-order GLCM-features that quantify local heterogeneity at the scale of a voxel; the features derived from high-order which quantify regional heterogeneity based on the neighborhood, the respective sizes and intensities of groups of voxels (Sollini et al. 2017). In the present work these parameters have been tested. In our series of patients, skewness was the only textural feature which showed a trend to differentiate benign from malignant nodules. Skewness, measuring the asymmetry of the probability distribution of intensity values about its mean, provides information about lesion heterogeneity. Considering the predictors selected in our population, SUV_max_ resulted in the highest specificity (81% versus 66%, 69%, and 31%) while TLG, skewness, as well as correlation_GLCM_ resulted better in sensitivity (100% versus 50% for SUV_max_ and 72% for both TLG and skewness). Therefore, based on its high negative predictive value, correlation_GLCM_ could be used to rule out a diagnosis of a malignant nodule, although its low specificity.

[^18^F]FDG uptake distribution has been associated with underlying physiopathological characteristics such as vascularization, perfusion, tumor aggressiveness, necrosis, hypoxia and gene expression (Tixier et al. 2014; Basu et al. 2011; Kunkel et al. 2003). Therefore, it is reasonable to hypothesize that significantly different values of PET image-derived heterogeneity quantification features may be observed in benign and malignant thyroid nodules, based on the different underlying physiopathological properties.

The parameters derived from routinely performed PET images may impact on clinical practice. In fact, up to 20% of FNA samples fall into category of insufficient/inadequate cytology, and approximately 15% to 30% of thyroid FNAs fall in an interpretive gray zone, in which the probability of malignancy is considered too high for watchful waiting but insufficient to propose a total thyroidectomy (Wise and Howard 2016; Bongiovanni et al. 2012; Cibas and Ali 2009; Nishino 2016). Aspirates in the “follicular neoplasm/suspicious for a follicular neoplasm” category (category TIR3B according to the update version of the SIAPEC-IAP (Nardi et al. 2014)) are typically associated with a 15%–30% risk of malignancy, therefore patients are generally referred for diagnostic thyroid lobectomy. Repeat FNA is the usual management for aspirates in the category of “atypia of undetermined significance/follicular lesion of undetermined significance” (TIR3A according to the update version of the SIAPEC-IAP (Nardi et al. 2014)) due to a 5%–15% risk of malignancy, with diagnostic lobectomy considered for nodules with repeatedly indeterminate FNA cytology (Nishino 2016). Therefore, the possibility to identify thyroid nodule with a high risk of malignancy, using texture analysis of conventional imaging on which the incidentaloma has been discovered, would have a great impact in clinical practice, determining better selection of patients proposed for FNA and guide patient management. The 2015 American Thyroid Association Management Guidelines (Haugen et al. 2016) recommend FNA in case of focal [^18^F]FDG uptake within a sonographically confirmed thyroid nodule ≥ 1 cm (strong recommendation, moderate-quality evidence). It is true that a focal [^18^F]FDG uptake within a thyroid nodule has an increased risk of malignancy, but it is also true that not all thyroid nodules characterized by a focal [^18^F]FDG uptake are malignant. Additionally, the number of passes for an adequate thyroid FNA can vary considerably, and a variety of factors, including operator’s skill, can influence its adequacy (Pitman et al. 2008). Therefore, the chance to identify through texture analysis patients who can benefit from additional workup for thyroid incidentaloma, could result in fewer FNA procedures and possibly fewer diagnostic thyroid lobectomies, decreasing consequently the cost for the healthcare system, and improving patients’ quality of life.

The present study has some limitations. Firstly, it is retrospective and evaluated a limited number of patients. In fact, only a small percentage of patients performed FNA and had a final diagnosis (12% and 11%, respectively) among all those for whom a focal [^18^F]FDG uptake was described in the PET/CT report. This is partly related to the fact that in our institution sonographers have a high expertise, thus cytological examinations are reserved only to patients presenting doubtful or suspected ultrasound findings; secondly to the fact that many patients who performed a PET/CT scan in our hospital were followed-up in other institutions. Therefore, we limited the analysis only to patients who performed the additional workup (i.e., ultrasound, FNA and histology) in our center. Conventional parameters and textural features were not tested within malignant nodules to differentiate thyroid cancer from other malignancies due to the disproportion of sample size in the two subsets of patients. Some features, including shape and size, GLCM, GLZLM, NGLDM, and GLRLM-based metrics, were available for approximately a half of cases, potentially underestimating the potentiality of this innovative approach. This limitation is related to the fact that the software used for texture doesn’t calculate features for volumes smaller than 64 voxels, as can be thyroid nodule. A validation of our results was not done. Finally, correction for the partial volume effect has not been performed.

## Conclusions

PET texture-derived features seem able to stratify the patients with thyroid incidentaloma identified on [^18^F]FDG-PET/CT scans, with respect to the risk of the diagnosis of a malignant thyroid nodule. Thus, the patients to be referred for FNA may be properly selected using post-processing techniques on routinely performed imaging on which the thyroid incidentaloma has been discovered.

## Additional file

Additional file 1: Table S1. Conventional PET parameters and textural features tabulated according FNA categories and final diagnosis. (DOCX 84 kb)

## Abbreviations

[^18^F]FDG: 2-deoxy-2-[^18^F]fluoro-D-glucose
3D: Tridimensional
AUC: Area under the curve
EAMN: European Association of Nuclear Medicine
EARL: EANM Research Ltd.
FNA: Fine-needle aspiration
GLCM: Gray-level co-occurrence matrix
GLRLM: Gray-level run-length matrix
GLZLM: Gray-level zone-length matrix
hCT: Calcitonin
Hist: Histogram
MTV: Metabolic tumor volume
NGLDM: Neighborhood gray-level different matrix
PET: Positron emission tomography
PET/CT: Positron emission tomography/computed tomography
ROC: Receiver operating characteristic
ROI: Region of interest
SIAPEC-IAP: Italian Society for Anatomic Pathology and Cytology joint with the Italian Division of the International Academy of Pathology
SUV_max_: Maximum standardized uptake value
TgAb: Anti-thyroglobulin antibodies
TLG: Total lesion glycolysis
TPOAb: Anti-thyroid peroxidase antibodies
TSH: Thyroid stimulating hormone
