# Supplementary material for: [18F]FDG-PET/CT texture analysis in thyroid incidentalomas: preliminary results
Source: Eur J Hybrid Imaging. 2017 Oct 12;1(1):3. doi: 10.1186/s41824-017-0009-8 (PMC5954705; doi:10.1186/s41824-017-0009-8)
Supplement: Supplementary file 1 — Conventional PET parameters and textural features tabulated according FNA categories and final diagnosis. (DOCX 84 kb) [file 41824_2017_9_MOESM1_ESM.docx]

**Additional file 1: Table 1. Conventional PET parameters and textural features tabulated according FNA categories and final diagnosis.**

| **Feature** | **FNA results** | | | | | | | **Final diagnosis** | | | | | |  |
| --- | --- | --- | --- | --- | --- | --- | --- | --- | --- | --- | --- | --- | --- | --- |
|  | **TIR2*** | **TIR3** | **TIR4** | | **TIR5** | **p** | | **Benign** | | **Malignant** | | **p** | |  |
| *Conventional^* | | | | | | | | | | | | | | |
| SUV_min_ | 2.1±1.3 | 2.5±1.3 | | 3.7±3.0 | 1.8±1.4 | 0.16 | | 2.1±1.2 | | 2.3±1.9 | | 0.67 | |  |
| SUV_mean_ | 4.1±3.5 | 6.5±6.4 | | 8.8±8.1 | 4.4±3.9 | 0.16 | | 4.1±3.2 | | 6.1±6.1 | | 0.35 | |  |
| SUV_std_ | 1.3±1.5 | 2.0±1.8 | | 3.7±3.6 | 1.6±1.7 | 0.09 | | 1.3±1.4 | | 2.2±2.4 | | 0.08 | |  |
| SUVmax | 7.3±6.5 | 12.3±12 | | 16.9±15 | 8.9±7.9 | 0.12 | | 7.4±6.1 | | 11.8±11.6 | | 0.01 | |  |
| SUVpeak1mL | 2.8±3.3 | 7.6±11.6 | | 4.1±5.8 | 4.2±6.1 | 0.39 | | 2.7±3.2 | | 6.0±8.7 | | 0.71 | |  |
| Volume | 11.9±22.5 | 114.7±243.1 | | 2.7±1.2 | 18.8±26.9 | 0.06 | | 10.7±20.5 | | 55.9±153.6 | | 0.23 | |  |
| TLG | 34.2±61.4 | 1961.0±5017.7 | | 18.8±10.7 | 66.7±110.2 | 0.09 | | 31.2±56.0 | | 804.2±3129.7 | | 0.95 | |  |
| *Histogram-based^* | | | | | | | | | | | | | | |
| Skewness | 0.7±0.5 | 0.5±0.5 | | 0.6±0.4 | 0.6±0.8 | 0.87 | | 0.7±0.4 | | 0.5±0.6 | | 0.02 | |  |
| Kurtosis | 3.0±1.2 | 2.7±0.8 | | 2.6±0.7 | 3.5±3.0 | 0.67 | | 2.9±1.2 | | 3.1±2.4 | | 0.41 | |  |
| Entropy | 1.4±0.3 | 1.5±0.1 | | 1.5±0.2 | 1.5±0.3 | 0.57 | | 1.4±0.3 | | 1.5±0.2 | | 0.94 | |  |
| Energy | 0.1±0.1 | 0.03±0.01 | | 0.04±0.01 | 0.04±0.03 | 0.58 | | 0.06±0.06 | | 0.04±0.03 | | 0.98 | |  |
| *Shape and size^#^* | | | | | | | | | | | | | | |
| Sphericity | 1.0±0.1 | 0.9±0.1 | | 1.0±0.2 | 0.9±0.1 | 0.82 | | 1.0±0.1 | | 0.9±0.1 | | 0.89 | |  |
| Compacity | 1.5±0.6 | 3.1±2.2 | | 1.0±0.1 | 1.6±0.5 | 0.03 | | 0.5±0.6 | | 2.0±1.5 | | 0.99 | |  |
| *GLCM-based^#^* | | | | | | | | | | | | | | |
| Homogeneity | 0.5±0.1 | 0.5±0.1 | | 0.3±0.1 | 0.5±0.2 | 0.41 | | 0.5±0.1 | | 0.5±0.2 | | 0.74 | |  |
| Energy | 0.04±0.03 | 0.08±0.13 | | 0.02±0.00 | 0.04±0.04 | 0.39 | | 0.03±0.03 | | 0.05±0.08 | | 0.97 | |  |
| Contrast | 12.4±15.2 | 17.3±20.4 | | 39.2±41.0 | 115.9±275.9 | 0.44 | | 12.6±14.7 | | 78.6±209.6 | | 0.99 | |  |
| Correlation | 0.5±0.2 | 0.6±0.1 | | 0.4±0.0 | 0.5±0.1 | 0.11 | | 0.5±0.2 | | 0.5±0.1 | | 0.99 | |  |
| Entropy | 1.7±0.4 | 1.8±0.2 | | 1.9±0.0 | 1.9±0.7 | 0.95 | | 1.8±0.4 | | 1.8±0.5 | | 0.80 | |  |
| Dissimilarity | 2.4±1.5 | 2.4±1.1 | | 4.7±3.0 | 4.8±7.4 | 0.51 | | 2.4±1.4 | | 4.1±5.7 | | 0.99 | |  |
| *NGLDM-based^#^* | | | | | | | | | | | | | | |
| Coarseness | 0.03±0.02 | 0.01±0.01 | | 0.05±0.02 | 0.03±0.03 | 0.23 | | 0.03±0.02 | | 0.03±0.03 | | 0.99 | |  |
| Contrast | 0.2±0.2 | 0.1±0.1 | | 0.4±0.3 | 0.4±0.7 | 0.47 | | 0.2±0.2 | | 0.3±0.5 | | 0.99 | |  |
| *GLRM-based^#^* | | | | | | | | | | | | | | |
| SRE | 0.9±0.1 | 0.9±0.0 | | 1.0±0.0 | 0.9±0.1 | 0.41 | 0.9±0.1 | | 0.9±0.1 | | | 0.30 | |  |
| LRE | 1.6±0.4 | 4.4±5.4 | | 1.2±0.1 | 1.8±0.8 | 0.10 | 1.6±0.4 | | 2.6±3.2 | | | 0.67 | |  |
| LGRE | 0.02±0.02 | 0.01±0.01 | | 0.01±0.01 | 0.02±0.02 | 0.54 | 0.02±0.02 | | 0.02±0.02 | | | 0.72 | |  |
| HGRE | 158.2±171.8 | 757.7±1293.3 | | 271.7±243.2 | 914.0±2071.6 | 0.49 | 159.6±166.1 | | 815.9±1681.4 | | | 0.99 | |  |
| SRLGE | 0.02±0.01 | 0.01±0.01 | | 0.01±0.01 | 0.02±0.01 | 0.58 | 0.02±0.01 | | 0.01±0.01 | | | 0.79 | |  |
| SRHGE | 147.6±166.0 | 590.1±980.4 | | 264.3±241.4 | 898.6±2055.4 | 0.21 | 149.1±160.5 | | 750.7±1614.8 | | | 0.74 | |  |
| LRLGE | 0.04±0.04 | 0.02±0.02 | | 0.01±0.01 | 0.05±0.05 | 0.48 | 0.04±0.04 | | 0.04±0.04 | | | 0.58 | |  |
| LRHGE | 212.0±196.4 | 12458.0±24568.3 | | 303.6±248.0 | 986.3±2131.9 | 0.12 | 213.0±189.8 | | 4759.6±14123.0 | | | 0.99 | |  |
| GLNU | 60.4±74.5 | 251.9±306.0 | | 9.7±8.6 | 70.1±89.3 | 0.07 | 58.2±72.5 | | 124.4±197.9 | | | 0.99 | |  |
| RLNU | 372.8±385.6 | 2747.1±4317.8 | | 102.2±48.2 | 373.3±250.5 | 0.07 | 367.9±373.1 | | 1125.9±2561.8 | | | 0.99 | |  |
| RP | 0.9±0.1 | 0.8±0.2 | | 0.9±0.0 | 0.9±0.1 | 0.17 | 0.9±0.1 | | 0.8±0.1 | | | 0.99 | |  |
| *GLZLM-based^#^* | | | | | | | | | | | | | | |
| SZE | 0.5±0.1 | 0.5±0.2 | | 0.6±0.2 | 0.5±0.2 | 0.63 | 0.5±0.1 | | | | 0.5±0.2 | | 0.74 |  |
| LZE | 768.3±1399.8 | 9078.3±14997.4 | | 15.7±17.9 | 2538.9±5397.9 | 0.13 | 722.4±1364.8 | | | | 4507.1±9441.7 | | 0.99 |  |
| LGZE | 0.02±0.02 | 0.01±0.01 | | 0.01±0.01 | 0.02±0.02 | 0.72 | 0.02±0.02 | | | | 0.02±0.02 | | 0.64 |  |
| HGZE | 161.8±151.0 | 690.9±1148.4 | | 272.6±259.4 | 916.7±2068.0 | 0.49 | 165.3±146.5 | | | | 792.3±1652.7 | | 0.99 |  |
| SZLGE | 0.01±0.01 | 0.01±0.01 | | 0.01±0.00 | 0.01±0.01 | 0.84 | 0.01±0.01 | | | | 0.01±0.01 | | 0.09 |  |
| SZHGE | 87.4±107.7 | 496.7±881.8 | | 180.7±198.7 | 779.9±1854.0 | 0.48 | 89.4±104.4 | | | | 640.6±1457.5 | | 0.99 |  |
| LZLGE | 29.4±68.9 | 23.0±36.2 | | 0.2±0.2 | 95.8±201.5 | 0.56 | 27.6±67.0 | | | | 63.5±155.3 | | 0.92 |  |
| LZHGE | 31679.6±42982.4 | 3223366.3±64221442.9 | | 2153.7±1345.3 | 74336.2±149907.2 | 0.10 | 29984.9±42074.7 | | | | 10784463.6±37088444.9 | | 0.78 |  |
| GLNU | 8.4±8.8 | 60.1±101.4 | | 3.8±2.2 | 7.2±5.5 | 0.09 | 8.3±8.5 | | | | 24.3±59.3 | | 0.21 |  |
| ZLNU | 35.5±61.5 | 980.2±1920.2 | | 17.4±9.4 | 79.9±133.1 | 0.12 | 35.2±59.4 | | | | 373.6±1102.9 | | 0.99 |  |
| ZP | 0.2±0.2 | 0.2±0.1 | | 0.5±0.3 | 0.3±0.3 | 0.38 | 0.2±0.2 | | | | 0.3±0.3 | | 0.99 |  |

*TIR2 category included also patients initially classified as TIR1

^Calculated for 50/50 patients; ^#^ calculated for 28/50 patients.
